# Exploring the effect of silver nanoparticle size and medium composition on uptake into pulmonary epithelial 16HBE14o-cells

**DOI:** 10.1007/s11051-016-3493-z

**Published:** 2016-07-02

**Authors:** Katja Kettler, Petra Krystek, Christina Giannakou, A. Jan Hendriks, Wim H. de Jong

**Affiliations:** Department of Environmental Science, Radboud University Nijmegen, Nijmegen, The Netherlands; Institute for Environmental Studies (IVM), VU University, Amsterdam, The Netherlands; IVAM UvA BV, Amsterdam, The Netherlands; National Institute for Public Health and the Environment (RIVM), Bilthoven, The Netherlands; Department of Toxicogenomics, Maastricht University, Maastricht, The Netherlands

**Keywords:** Nanoparticle, Uptake kinetics, Size dependence, Internal cellular concentration, Serum proteins, Health and safety effects, Biomedicine

## Abstract

The increasing number of nanotechnology products on the market poses increasing human health risks by particle exposures. Adverse effects of silver nanoparticles (AgNPs) in various cell lines have been measured based on exposure dose after a fixed time point, but NP uptake kinetics and the time-dependent internal cellular concentration are often not considered. Even though knowledge about relevant timescales for NP uptake is essential, e.g. for time- and cost-effective risk assessment through modelling, insufficient data are available. Therefore, the authors examined uptake rates for three different AgNP sizes (20, 50 and 75 nm) and two tissue culture medium compositions (with and without foetal calf serum, FCS) under realistic exposure concentrations in pulmonary epithelial 16HBE14o-cells. The quantification of Ag in cells was carried out by high-resolution inductively coupled plasma mass spectrometry. We show for the first time that uptake kinetics of AgNPs into 16HBE14o-cells was highly influenced by medium composition. Uptake into cells was higher in medium without FCS, reaching approximately twice the concentration after 24 h than in medium supplemented with FCS, showing highest uptake for 50-nm AgNPs when expressed on a mass basis. This optimum shifts to 20 nm on a number basis, stressing the importance of the measurand in which results are presented. The importance of our research identifies that not just the uptake after a certain time point should be considered as dose but also the process of uptake (timing) might need to be considered when studying the mechanism of toxicity of nanoparticles.

**Electronic supplementary material:**

The online version of this article (doi:10.1007/s11051-016-3493-z) contains supplementary material, which is available to authorized users.

## Introduction

Nanoparticles (NPs) are used in increasing amounts and number of applications (Hendren et al. 2013; Nowack and Bucheli 2007) due to their unique physical and chemical properties. Consumer products with NPs include toothpaste, wound dressings, antibacterial towels and sportswear, as well as in deodorants and in sprays for textiles and shoes (Kuiken et al. 2014; Oomen et al. 2011; Vance et al. 2015; Wijnhoven et al. 2011). Of special interest for consumer products, medical applications and industry are silver nanoparticles (AgNPs), attributable, besides others, to their antimicrobial properties. Several in vitro studies showed that AgNPs affect various cell lines, including phagocytizing cell like THP-1-derived macrophages, and serious concerns about toxicological and environmental effects have been raised (Foldbjerg et al. 2009; Haase et al. 2011; Park et al. 2011; Singh and Ramarao 2012).

So, it can be concluded that adverse effects can occur after contact with NPs. Exposure routes include dermal, intestinal and inhalation uptake, whereas injection may be used for nanomedicinal products. Inhalation of AgNPs (aerosols) is possible by consumers during product use (e.g. spray applications) or by workers during the production and processing of NPs (Maynard and Kuempel 2005). Also for AgNPs, respiratory toxicity was reported in various animal studies (Braakhuis et al. 2016a; Herzog et al. 2013; Ji et al. 2007; Seiffert et al. 2015; Silva et al. 2015). On the long term, the product containing the NPs may end up in the environment through wash off, posing a potential risk to human health and biota (Christensen et al. 2010). Unfortunately, effects are often measured after a single fixed time point based on exposure dose, neglecting uptake kinetics and therefore time-dependent internal cellular concentration. NP properties, such as size, influence uptake as shown for various cell lines in vitro. Unfortunately, uptake was detected only after a fixed time point (He et al. 2010; Jiang et al. 2008; Lu et al. 2009; Rejman et al. 2004; Wang et al. 2010).

Small NPs can enter cells after contact via one of the four exposure routes through a fundamental biological process called endocytosis as previously summarized (Kettler et al. 2014). Particulate matters, such as proteins and other nutrients, are taken up into eukaryotic cells via enclosure by the cell membrane (Conner and Schmid 2003). Endocytosis is a form of active transport. Endocytosis is usually employed by non-phagocytic cells like 16HBE to take up biomolecules from the environment. The cell membrane forms itself around the molecules/particle to be taken up, and the size of the resulting vesicles depends on the presence of ligands and the protein involved in vesicle formation (Doherty and McMahon 2009). Macropinocytosis is a non-specific mechanism by which the cell surrounding fluid and its content are taken up in a concentration as present with vesicles from 100 nm to 5 µm in size (Basu 1984). The other pathways are receptor mediated, and uptake occurs after binding of a ligand to specific receptors in the cell surface, which results in uptake of the ligand in higher concentration than in the medium. These pathways are further distinguished by the protein involved in vesicle formation. Vesicles in receptor-mediated endocytosis reach sizes up to roughly 80 nm in diameter (i.d.) (caveolae mediated) (Hirota and Terada 2012) or 120 nm i.d. (clathrin mediated) (Conner and Schmid 2003; Hirota and Terada 2012). Internalized NPs may cause the production of reactive oxygen species due to their effects on mitochondrial respiration (Xia et al. 2008), and antioxidant species might be depleted in the cell (Park et al. 2008). Metals in the form of NPs may employ this mechanism, and metal ions then may enter cells by a so-called Trojan horse effect. Especially in the case of soluble metal particles, this can result in high levels of the metal in various ionic species inside the cell resulting in further reactions (Limbach et al. 2007; Luoma 2008). By this mechanism, AgNPs can enter cells and release toxic Ag ions inside the cell “bypassing its barriers to “normal”-sized Ag and then releasing Ag ions that damage cell machinery” (Lubick 2008), thereby increasing the intracellular bioavailability of Ag. It was shown that AgNPs can induce a higher toxic damage than Ag ions (Lubick 2008; Park et al. 2011). As summarized previously, several experimental conditions may influence NP uptake (Kettler et al. 2014). In particular, the presence or absence of serum proteins has been shown to have great effects through the formation of a so-called protein corona on the NPs surface. Uptake can be increased, decreased or remain the same, depending on the type of protein present (Ikada and Tabata 1986; Krystek et al. 2015; Nagayama et al. 2007; Sbarra and Karnovsky 1959). In this study, we aim to determine rates of uptake for different AgNP sizes and cell culture medium with and without serum proteins, therefore exposing 16HBE14o-cells to NP for different periods of time over 24 h. We apply NP concentrations of 0.01 µg Ag/mL that represent realistic concentrations for short-term exposures (Gangwal et al. 2011). This concentration is much lower, namely by a factor of up to 10,000, than in some of the existing literature where uptake rates have been determined (Huang et al. 2010; Shapero et al. 2010). These data will deliver new insights into the effect of NP size on their uptake rate and are required to model and predict NP uptake rates based on easily measurable NP properties. Combining empirical data and modelling will be an indispensable tool for time- and cost-effective risk assessment of NPs.

## Materials and methods

### Nanomaterials

For non-phagocytic cells like 16HBE14o, a maximum vesicle size of 80–120 nm has been found (Conner and Schmid 2003; Hirota and Terada 2012); therefore, three differently sized AgNPs were investigated in this study: 20-nm citrate BioPure™ Silver, 50-nm citrate BioPure™ Silver and 75-nm citrate BioPure™ Silver were purchased from NanoComposix Inc (San Diego, CA) in the form of stock dispersion with concentrations of 1 mg/mL in aqueous 2 mM citrate buffer. The supplier provided detailed information about the NPs characteristics as summarized in Table 1. The diameter was detected by the manufacturer NanoComposix Inc using transmission electron microscopy (TEM) (JEOL 1010 transmission electron microscope), and the hydrodynamic diameter through dynamic light scattering (DLS) (Malvern Zetasizer Nano ZS). The material for TEM imaging is prepared by drying nanoparticles on a copper grid; samples for DSL measurements are undiluted in case of 20-nm AgNPs and diluted with water by a factor of 10 and a factor of 20 for 50- and 75-nm AgNPs, respectively. According to the supplied information, the NPs are stable for at least 1 year; however, prolonged exposure to light may change the material size. Accordingly, the NPs were always kept out of light as much as possible, but light exposure could not be completely prevented during handling.

**Table 1:** AgNP characteristics as provided by the manufacturer NanoComposix Inc

AgNP product (nm)	Average diameter (nm)	Coefficient of variation (%)	SD (nm)	Average hydrodynamic diameter (nm)	Zeta potential (mV)	Mass concentration (mg Ag/mL)
20	19.6	8.1	1.6	24.0	−30.4	1.09
50	53.5	8.3	4.4	56.0	−53.6	0.95
75	74.8	5.8	4.3	78.5	−37.6	0.99

### Preparation of AgNPs dispersions

Due to the significant effects, serum proteins present in tissue culture growth medium may have on uptake, these uptake studies were conducted with two types of medium, with foetal calf serum (+FCS) and without (w/o) the addition of FCS (Sbarra and Karnovsky 1959; Ikada and Tabata 1986; Nagayama et al. 2007). The dilutions of AgNPs dispersions were performed in complete tissue culture medium (see below), either without or with FCS, prior to exposure. Final exposure concentrations of 0.01 µg Ag/mL were obtained in several steps and pre-dilutions: Per 1 mL medium, 10 µL of the NP dispersion was added for the first pre-dilution and mixed thoroughly. This pre-dilution was further diluted in three steps to reach the exposure concentration of 0.01 µg Ag/mL. Two hundred microliters of the final dilution was added to each well. We used concentrations of 0.01 µg/mL at which no change in metabolic toxicity has been shown in 16HBE14o-cells for NPs of the same type, from the same manufacturer and same sizes (20 and 50 nm) in the absence of FCS in order to study uptake in uncompromised cells (Braakhuis et al. 2016b).

### Cells and cell culture conditions

The human pulmonary epithelial cell line 16HBE14o (kindly provided by Dr. Gruenert, USA) was used in this study. 16HBE14o cells were cultured in Dulbecco’s Modified Eagle Medium/Nutrient Mixture F-12 + GlutaMAX (DMEM/F-12, Gibco, the Netherlands), supplemented with antibiotics (1 % penicillin–streptomycin (Pen/Strep), Gibco, the Netherlands) and 5 % foetal calf serum (FCS, Gibco, the Netherlands) designated complete medium. When the cells reached an 80–100 % confluent monolayer, they were subcultured, usually 1:5 once a week in new 75-mL tissue culture flasks (CellStar^®^, Greiner Bio-one, the Netherlands). 16HBE14o-cells were harvested enzymatically using trypsin solution (trypsin–EDTA (0.05 %) and phenol red, Gibco, the Netherlands), counted, diluted and seeded into 96-well plates at cell densities of 60,000–65,000 cells/well 24 h before exposure to allow them to rest and to reach 80–90 % confluence at the day of exposure. Cells were constantly incubated at 37 °C and 5 % CO_2_ atmosphere.

### Cellular uptake of AgNPs

Cells were seeded in 96-well tissue culture plates as described in “cell and cell culture conditions” section and exposed to 200 µL of 0.01 µg/mL AgNP dispersions. The total exposure times were 0, 2, 4, 8, 12 and 24 h. At the end of exposure, cells were thoroughly washed with Dulbecco’s phosphate-buffered saline (DPBS, without calcium, magnesium, phenol red, Gibco, the Netherlands) twice to remove loosely attached Ag ions and/or NPs from the cell membrane. Two hundred microliters of trypsin solution was added to detach the cells from the bottom of the well. Through pipetting, it was made sure that all cells were detached, and successful detachment was confirmed by optical inspection with a light microscope (magnification 50×). Subsequently, the sample was transferred to 15-mL polystyrene centrifuge tubes (Corning Life Sciences, the Netherlands) to be stored at 4 °C until their measurement of tightly attached and internalized NPs. The samples were treated with 2 mL aqua regia (nitric acid and ultrapure hydrochloric acid, 1:3, both from Merck, Germany) and further diluted with ultrapure water to a final volume of 10 mL prior to the determination of the total Ag concentration (particle associated as well as ionic Ag) by HR-ICP-MS-type ELEMENT XR, Thermo Fisher Scientific, Bremen, Germany. The sample introduction systems consisted of a concentric nebulizer, a cyclone spray chamber and nickel cones (all from the instrument supplier). For the quantification, external calibration with internal standard correction (Rhodium as ^103^Rh^+^) was applied. Sample pre-treatment and analysis were carried out in a clean room facility class 10,000. Each experiment was conducted independently two times for 50- and 75-nm AgNPs and once for 20-nm AgNPs.

### Statistical evaluation and calculations

For the determination of uptake rate constants c and elimination rate constants k, experimental results were evaluated with Microsoft Excel 2010 and the corresponding application “Solver” according to the one compartment model using Eq. 1. Solver is a tool that determines the optimal value for variables, here *c* and *k*, within given limits in order to minimize differences between experimental and model data.

1 $$c\left( t \right) = \frac{c}{k} \cdot \left( {1 - {\text{e}}^{ - k \cdot t} } \right)$$

where *t* is the time of exposure in hours; *c* is the uptake rate constant in ng Ag well^−1^ d^−1^; and *k* is the elimination rate constant in ng Ag well^−1^ d^−1^. Values for *k* were set to a minimum of 10^−8^ in order to avoid divisions by zero during the calculations, no other limits are set. Ag concentrations were standardized to a starting concentration of zero by subtracting the initial concentration in the cells from the concentrations in the cells at the later time point.

To convert the amount of Ag/well as detected by HR-ICP-MS to assumed spherical NP numbers/well (NP No/well), the formulas according to Chithrani et al. (2006) were used.

## Results

**Fig. 1:**
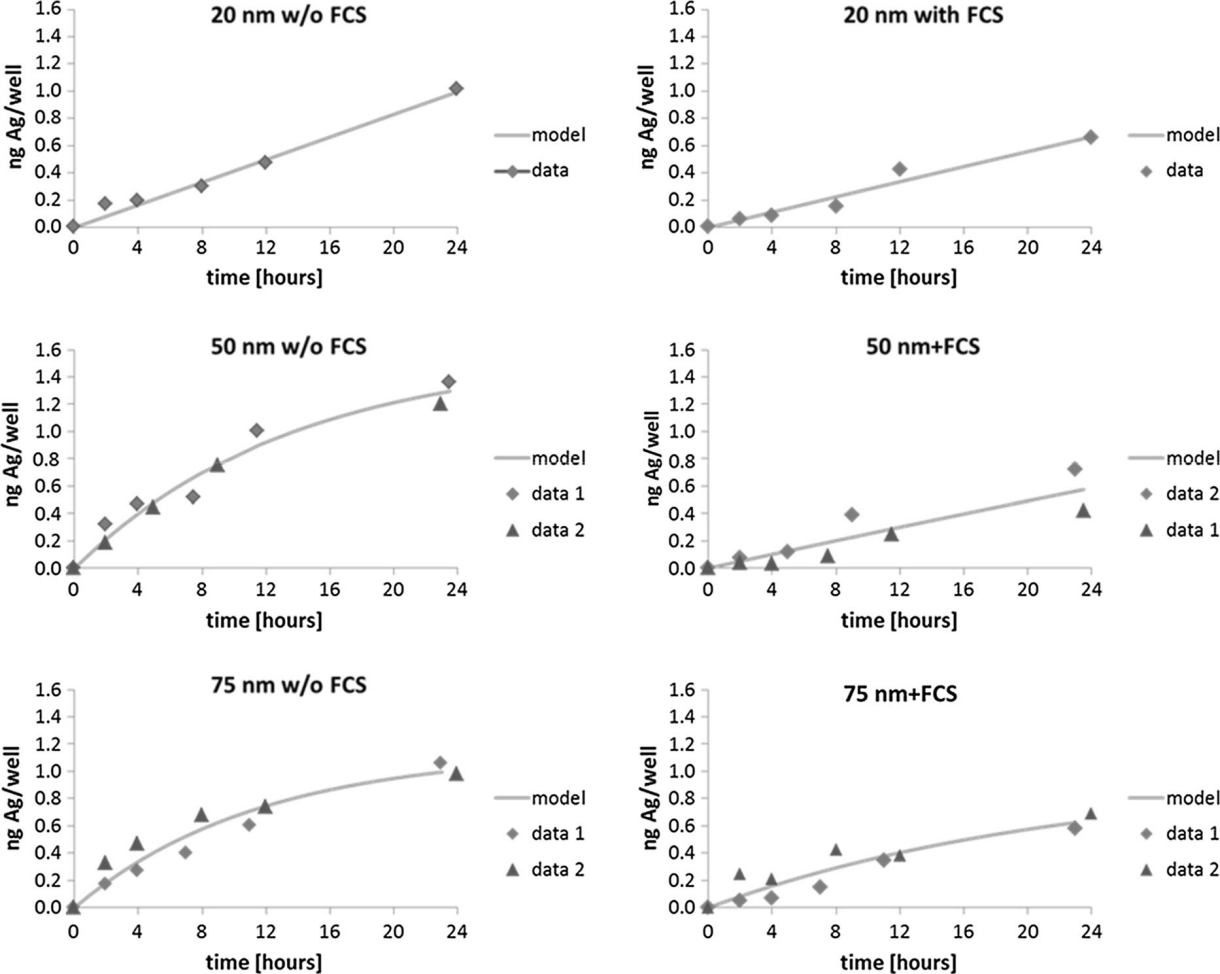
Ag amount in cells on mass basis with the fitted line for three NP sizes and medium without and with the addition of foetal calf serum based on one and two independent experiments for 20-, 50- and 75-nm particles, respectively. *Note*: Due to logistic aspects, the time points at which the samples have been obtained are slightly shifted sometimes (maximum 1 h)

The original result of each sample point as well as the model uptake curves is given in Fig. 1. Uptake did not clearly level off during the time course for all samples. For the 50- and 75-nm samples without FCS, the slope of the uptake curve clearly became less steep with time; the curve of the 75-nm samples with FCS levelled off slightly. In our set-up the uptake of 50 AgNPs was higher for medium without FCS than in medium supplemented with FCS, reaching approximately twice the maximum concentration after 24 h of exposure. Uptake was also faster, as represented by the significantly different 95 % confidence interval of the uptake rate constant c of 1.1 × 10^−1^–1.2 × 10^−1^ and 6.9 × 10^−3^–5.2 × 10^−2^ ng Ag well^−1^ d^−1^ without and with FCS, respectively. For 20- and 75-nm AgNPs, the same trend was observed but not as pronounced, and the difference in uptake rates is statistically not significant.

No differences in uptake, expressed as ng Ag per well, for the three NP sizes could be observed in the presence of FCS (Table 2); their uptake rate constants were not significantly different. The mean of all three AgNP sizes fell within the same narrow range.

**Table 2:** Overview of the uptake curves based on mass (ng) Ag

NP size (nm), medium type	c	Std. dev. of c	95 % CI of c
20 w/o FCS	4.2 × 10^−2^	n.a.	n.a.
50 w/o FCS	1.1 × 10^−1^	4.0 × 10^−3^	1.1 × 10^−1^–1.2 × 10^−1^
75 w/o FCS	1.1 × 10^−1^	6.10 × 10^−2^	2.7 × 10^−2^–2.0 × 10^−1^
20+FCS	2.8 × 10^−2^	n.a.	n.a.
50+FCS	2.9 × 10^−2^	1.6 × 10^−2^	6.9 × 10^−3^–5.2 × 10^−2^
75+FCS	4.8 × 10^−2^	2.9 × 10^−2^	7.5 × 10^−3^–8.8 × 10^−2^

Average uptake rate constants c, their standard deviation (Std. dev) and 95 % confidence interval (CI) based on NP mass, all given in ng Ag well^−1^ d^−1^

*n*.*a*. not analysed

Uptake in medium without FCS was faster, and a size effect regarding the concentration at the latest time point could be seen (Fig. 1, Figure S1), although there is large variation between replicates. Uptake expressed as mass was highest for AgNPs with a size of 50 nm, reaching internal cell concentrations of approximately 65 % of the nominal concentration, followed by AgNPs of 75 and of 20 nm, each with roughly 50 % Ag uptake. For the latter AgNP size, only a single experiment was conducted, yet it did not fall into the confidence interval of the 50-nm samples. The uptake and elimination rates have been determined as presented in Tables 2 and S1, respectively. For medium supplemented with FCS, no difference in uptake rates could be detected (range 0.03–0.05 ng Ag well^−1^ d^−1^). The observed trend of highest uptake for 50-nm AgNPs was not reflected in the uptake rate, as the confidence intervals overlap for 50- and 75-nm AgNPs.

**Fig. 2:**
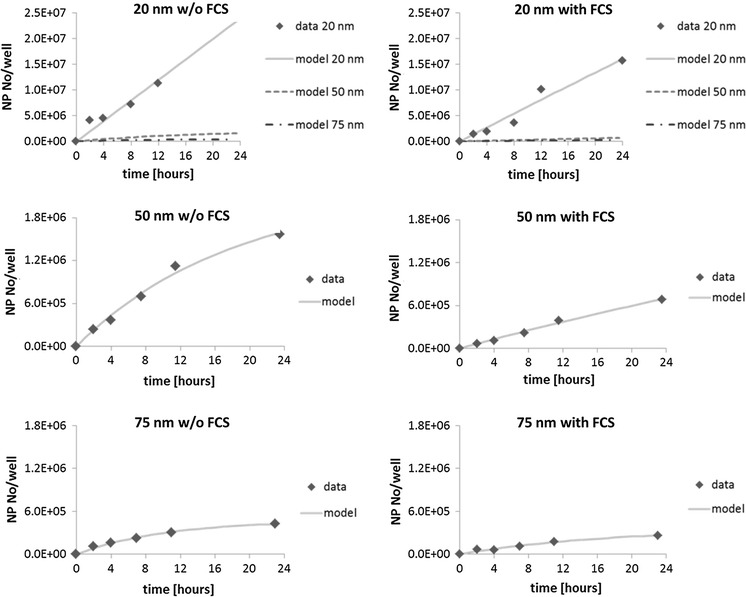
Average Ag content in cells on a NP number basis calculated from the independent mass-based values for three NP sizes and medium without and with foetal calf serum as shown in Fig. 1. The two *top graphs* show model data for all three NP sizes for easy comparison and calculated average NP numbers for 20-nm AgNPs. The four graphs on the *bottom* show the average Ag content and the corresponding model data for 50- and 75-nm AgNPs in more detail. *Note*: Due to logistic aspects, the time points at which the samples have been obtained are slightly shifted sometimes (maximum 1 h); different scaling on the *y* axes

A size optimum based on particle numbers does not necessarily lead to the same uptake optimum regarding NP mass (Lévy et al. 2010). To test whether this was the case here as well, the values were converted to particle numbers and their uptake rates compared. In this case, a clear difference, represented by the slope, was observed (Fig. 2). The uptake rate, expressed as particle numbers per well per day, differed significantly for 20-nm AgNPs from the rate of 50- and 75-nm AgNPs, both in the absence and presence of FCS (Table 3). No difference in the uptake rate in the presence of FCS could be observed for 50- and 75-nm AgNPs, while the rate was higher for 50-nm AgNPs than 75-nm AgNPs in the absence of FCS.

**Table 3:** Overview of the uptake curves based on NP numbers (NP no.)

NP size (nm), medium type	c	Std. dev. of c	95 % CI of c
20 w/o FCS	1.0 × 10^6^	n.a.	n.a.
50 w/o FCS	1.4 × 10^5^	4.7 × 10^3^	1.3 × 10^5^–1.4 × 10^5^
75 w/o FCS	4.8 × 10^4^	2.6 × 10^4^	1.2 × 10^4^–8.4 × 10^4^
20+FCS	6.8 × 10^5^	n.a.	n.a.
50+FCS	3.5 × 10^4^	1.9 × 10^4^	8.2 × 10^3^–6.1 × 10^4^
75+FCS	2.1 × 10^4^	1.3 × 10^4^	3.2 × 10^3^–3.8 × 10^4^

Average uptake rate constants c, their standard deviation (Std. dev) and 95 % confidence interval (CI) based on NP number, all given in NP number well^−1^ d^−1^

*n*.*a*. not analysed

## Discussion

Our results show that the tissue culture medium composition in terms of the presence or absence of foetal calf serum affects NP uptake significantly, as confirmed by the literature (Sbarra and Karnovsky 1959; Ikada and Tabata 1986; Nagayama et al. 2007). When no FCS is present, final concentrations after 24 h expressed on a mass basis are higher for 50-nm AgNPs than for 20- and 75-nm AgNPs. Internal cellular concentrations, expressed as a percentage of the nominal added concentrations, reached roughly 50 % (20-, 75-nm NPs) and 65 % (50-nm NPs). FCS decreases the amount of silver taken up on a mass basis by roughly 25, 54 and 40 % for 20-, 50- and 75-nm AgNPs, respectively. A possible explanation is the formation of a protein corona around the AgNPs in the medium with FCS that reduces their uptake. Anionic bovine serum albumin, for example, showed repulsive interaction with the negatively charged cell membrane (Zhu et al. 2012). Recently, it was reported that also for the uptake of fluorescent silica NPs, an increase in serum concentration in the tissue culture medium resulted in a decrease in cellular uptake in human mesenchymal stem cells (hMSC) (Catalano et al. 2015). We show the importance to consider various medium compositions in toxicity studies, yet further confirmation for NPs of different charge and various types of serum proteins is needed. The higher uptake of 50-nm particles on mass basis in absence of FCS might be partially attributed to the more negative zeta potential of these AgNPs. At first, a higher uptake of more negatively charged NPs across the overall negatively charged cell membrane seems illogical due to repulsion. Yet, an early study already reports that positively charged patches on the cell surface exist (Ghinea and Simionescu 1986). The positively charged residues “seem to be accumulated in coated pits” (Ghitescu and Fixman 1984) and could, therefore, be responsible for the binding and uptake of negatively charged NPs. An increase in the absolute zeta potential usually leads to increased NP uptake in comparison with less charged NPs of the same size (Kettler et al. 2014). Whether size or charge has the greater influence on uptake remains unknown up to date due to a limited number of uptake kinetics studies and a lack in availability of NPs with proper characteristics to study these phenomena. This stresses the need for commercially available NPs with only one property changed at a time or, ideally, that reference materials were made available.

In contrast to the uptake studies by Chithrani et al. (2006) over 10 h, uptake did not always clearly level off in time in our set-up (Fig. 1). This might be attributed to the different elemental NP composition (gold vs. Ag) (Ahsan et al. 2002) and different cell lines (HeLa vs. 16HBE14o) as summarized by us previously (Kettler et al. 2014); uptake of NPs also shows a concentration dependency (Asharani et al. 2009; Chithrani et al. 2006; Giljohann et al. 2007; Limbach et al. 2007). Giljohann et al. (2007) showed that uptake does not necessarily relate to exposure concentrations in a linear fashion. Catalano et al. (2015) observed a levelling-off of the NP uptake in hMSC in the presence of serum, whereas such a levelling-off was not present in the testing conditions without serum over a time course of only 6 h, what makes a comparison with our results difficult. Yet, our results show the opposite trend also for such short incubation times. Overall, our results show that uptake kinetics differ per NP and should be taken into account in toxicity studies.

Although generally mass is used as dosing parameter (it is also highly practical and convenient) for nanoparticles, the unit that reacts with a cell is the whole particle. When mass is used as a dose for a soluble chemical, this translates to a number of molecules reacting with a cell or cell receptors. Although nanoparticles also are composed of molecules and these molecules can react with cell receptors, due to its form (e.g. spheric, cube or tube like) not all individual molecules can react with a cell or cell receptor. It is the whole particle that can be composed of a few but also many molecules depending on the size (and size distribution) of the investigated nanoparticle. So, a dose in mass, for nanoparticles, cannot be translated in a dose of molecules interacting with a cell or cell receptor. The dose in nanotoxicology is still under debate and can be dependent on the toxic parameter investigated. For example, for lung inflammation the total surface area was found to be a better dose description than mass (Braakhuis et al. 2016a). Also, it is debated whether for in vitro cytotoxicity experiments, the dose in the medium should be used (in concentration of mass, surface area or particle number) or the dose as what the cells see or take up (DeLoid et al. 2015; Teeguarden et al. 2007).

It is also clearly shown that the measurand, in which results are presented, plays a very important role in the interpretation of the results. While in most cases, no significant difference in uptake rate can be detected on a mass basis, there is a very clear effect of particle size on the uptake rate of 20-nm AgNPs when the results are presented on a number of particle basis (Fig. 2). Uptake of smaller particles is faster than of larger particles independent of the culture medium composition; except for 50- and 75-nm AgNPs + FCS, no significant difference was observed, but the total mass is equally fast, except for 20- and 50-nm AgNPs w/o FCS. The size optimum, which relates to the highest uptake rate, of 50 nm is only in good agreement with other studies when expressed on a mass basis, while it is not when expressed as particle number (Chithrani et al. 2006; Shapero et al. 2010). In contrast to us, those studies found the highest uptake rate at 50 nm based on a particle number basis in various cell lines. Few other studies exist where uptake rates have been determined, yet they are difficult to compare to our studies. The reason for this is the difference in the used NP sizes. The following results are all converted to and reported on NP number basis. Uptake rates are found to be highest for 44 nm, followed by 114 and 33 nm (Varela et al. 2012). The other researchers find 32 nm to show highest uptake rates, followed by 70, 102, and 118 nm, respectively (Huang et al. 2010). They originally report their results on mass basis; then, the order is as follows: 102, 70, 118 and 32 nm. No clear pattern can be observed on mass basis. Only one of the few studies that also determine uptake rates is in agreement with our results. Expressed on NP number basis, uptake rates are also highest for 20-nm particles, followed by 40 nm and lowest for 100 nm (Wang et al. 2009). Therefore, no conclusions about uptake optima based on particle number basis can be directly drawn even though all studied cell lines can utilize the mechanism of endocytosis just as 16HBE cells. The exact uptake mechanism for AgNPs into 16HBE cells used in our experiments is unknown because the determination of the exact uptake mechanism is very complex. It requires the determination of the exact concentration and incubation time for various inhibitors and has to be adjusted for each cell line separately (Kuhn et al. 2014). Pinocytosis can be excluded as the uptake mechanism because it is a non-saturable process and saturation is clearly observed (Fröhlich 2012).

The non-uniform way of presenting results may lead to misinterpretation and false conclusion about agreement between studies, as criticized by Lévy et al. (2010). They showed that an optimum based on the number of particles per cell does not necessarily lead to the same optimum in terms of mass. This was also nicely shown by Yue et al. (2010), who graphically showed that the size optimum depends on the measurand (number of particles per cell, particle volume per cell and particle surface area per cell).

We therefore recommend to pay close attention to the measurand given in various studies and would find it helpful if the optimum was given also in different measurands in one study for comparability between studies. Often important data for such conversions are not directly accessible in publications, e.g. it might be difficult to calculate the number of atoms per particle because only the total diameter is known but not the thickness of a coating.

## Conclusion

The present study shows that the medium composition (absence or presence of FCS) has a large effect on the speed of uptake and therefore the maximum concentration after 24 h of exposure. This should be taken into consideration in (toxicity) studies, where uptake and internal concentration play a crucial role in the understanding of the mechanism of the observed effects. Uptake in medium without FCS shows a trend towards higher uptake for 50-nm AgNPs than 75- and 20-nm AgNPs on a mass basis. This uptake optimum shifts to 20-nm AgNPs when expressed on a AgNP number basis in contrast to other results reported in the literature. For comparability between studies, direct conversion of uptake expressed as NP number and mass in one publication is advantageous; sometimes, the necessary information to do so oneself is not directly available in the publication. Future developments will greatly benefit from the use of reference materials. Since the large and continuously growing number of engineered NPs cannot all be covered experimentally, comparability between studies would possibly help to close knowledge gaps and allow for extrapolation regarding uptake between different NPs by modelling. Modelling will be an indispensable tool to cover the large and continuously growing number of engineered NPs and to predict the uptake of untested NPs.

## Electronic supplementary material

Below is the link to the electronic supplementary material.

Figure S1 Ag amount on bass basis with 95 % confidence intervals (TIFF 162 kb)

Table S1 Overview of the elimination rates based on mass [ng] Ag; Table S2 Overview of the elimination rates based on NP numbers [NP No]. (PDF 31 kb)
