# Supplementary figures and images for: Exploring the effect of silver nanoparticle size and medium composition on uptake into pulmonary epithelial 16HBE14o-cells
Source: J Nanopart Res. 2016 Jul 2;18:182. doi: 10.1007/s11051-016-3493-z (PMC4930793; doi:10.1007/s11051-016-3493-z)

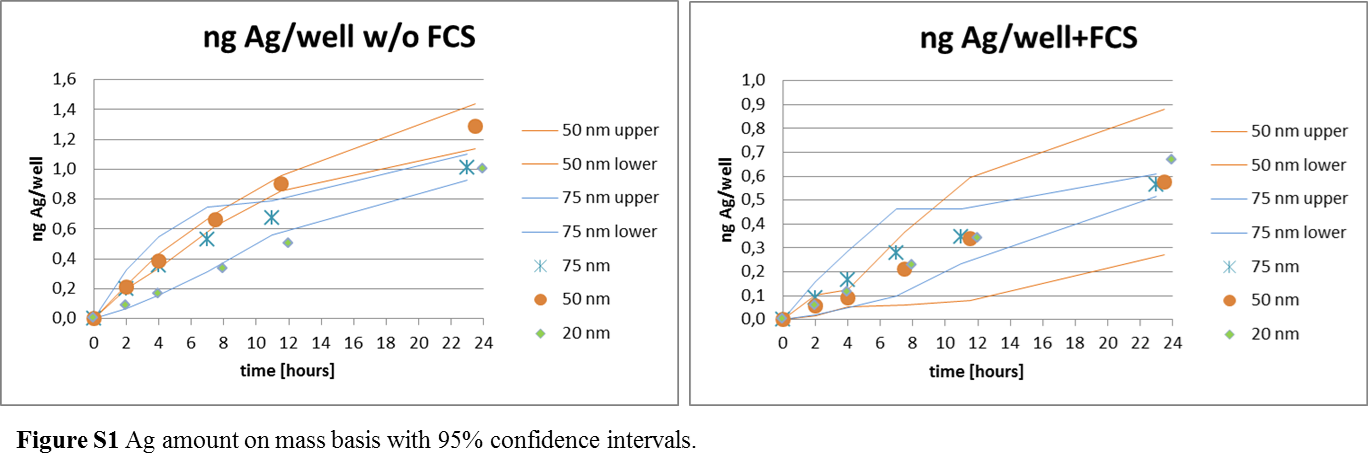

Supplement: Supplementary file 1 — Ag amount on bass basis with 95 % confidence intervals (TIFF 162 kb) [file 11051_2016_3493_MOESM1_ESM.tif]
