# Supplementary material for: Exploring the effect of silver nanoparticle size and medium composition on uptake into pulmonary epithelial 16HBE14o-cells
Source: J Nanopart Res. 2016 Jul 2;18:182. doi: 10.1007/s11051-016-3493-z (PMC4930793; doi:10.1007/s11051-016-3493-z)
Supplement: Supplementary file 2 — Overview of the elimination rates based on mass [ng] Ag; Table S2 Overview of the elimination rates based on NP numbers [NP No]. (PDF 31 kb) [file 11051_2016_3493_MOESM2_ESM.pdf]

Table S1 Overview of the elimination rates based on mass [ng] Ag

Average elimination rate constants k, their standard deviation (Std. dev) and 95 % confidence Interval (CI) based on NP mass, all given in ng Ag$\cdot$well$^{-1}\cdot$d^{-1}.

NP size [nm], medium type	k	Std. dev. of k	95% CI of k
20 w/o FCS	$1.0\cdot 10^{-7}$	n.a.	n.a.
50 w/o FCS	$7.3\cdot 10^{-2}$	$5.3\cdot 10^{-3}$	$6.5\cdot 10^{-2}$-$8.0\cdot 10^{-2}$
75 w/o FCS	$9.9\cdot 10^{-2}$	$8.9\cdot 10^{-2}$	$-2.4\cdot 10^{-2}$-$2.2\cdot 10^{-1}$
20+FCS	$1.9\cdot 10^{-3}$	n.a.	n.a.
50+FCS	$1.1\cdot 10^{-2}$	$1.6\cdot 10^{-2}$	$-1.1\cdot 10^{-2}$-$3.3\cdot 10^{-2}$
75+FCS	$4.9\cdot 10^{-2}$	$6.2\cdot 10^{-2}$	$-3.6\cdot 10^{-2}$-$1.3\cdot 10^{-1}$

n.a.: not analysed

Table S2 Overview of the elimination rates based on NP numbers [NP No]

Average elimination rate constants k, their standard deviation (Std. dev) and 95 % confidence Interval (CI) based on NP number, all given in NP number$\cdot$well$^{-1}\cdot$d^{-1}.

NP size [nm], medium type	k	Std. dev. of k	95 % CI of k
20 w/o FCS	$1\cdot 10^{-8}$	n.a.	n.a.
50 w/o FCS	$7.3\cdot 10^{-2}$	$5.3\cdot 10^{-3}$	$6.5\cdot 10^{-2}$-$8.0\cdot 10^{-2}$
75 w/o FCS	$1.0\cdot 10^{-1}$	$8.9\cdot 10^{-2}$	$-2.4\cdot 10^{-2}$-$2.2\cdot 10^{-1}$
20+FCS	$1.9\cdot 10^{-3}$	n.a.	n.a.
50+FCS	$1.1\cdot 10^{-2}$	$1.6\cdot 10^{-2}$	$-1.1\cdot 10^{-2}$-$3.3\cdot 10^{-2}$
75+FCS	$4.9\cdot 10^{-2}$	$6.2\cdot 10^{-2}$	$-3.6\cdot 10^{-2}$-$1.4\cdot 10^{-1}$

n.a.: not analysed
